# Complete Genome Sequence of *Anoxybacillus flavithermus* TNO-09.006, a Thermophilic Sporeformer Associated with a Dairy-Processing Environment

**DOI:** 10.1128/genomeA.00010-13

**Published:** 2013-02-28

**Authors:** Martien P. M. Caspers, Jos Boekhorst, Tjakko Abee, Roland J. Siezen, Remco Kort

**Affiliations:** a Top Institute (TI) Food and Nutrition, Wageningen, The Netherlands; b TNO, Microbiology and Systems Biology, Zeist, The Netherlands; c NIZO Food Research, Ede, The Netherlands; d Laboratory of Food Microbiology, Wageningen University and Research Centre, Wageningen, The Netherlands; e Netherlands Bioinformatics Centre, Nijmegen, The Netherlands; f Molecular Cell Physiology, Vrije Universiteit (VU) University Amsterdam, Amsterdam, The Netherlands; g Microbial Bioinformatics, Ede, The Netherlands

## Abstract

Spores of thermophilic spore-forming bacteria are a common cause of contamination in dairy products. We isolated the thermophilic strain *Anoxybacillus flavithermus* TNO-09.006 from a milk-processing plant, and we report the complete genome of this isolate consisting of a single chromosome of 2.65 Mb.

## GENOME ANNOUNCEMENT

One of the regular problems in the production of dairy concentrates is contamination by heat-resistant spores from the biofilms of thermophilic bacteria of the genera *Anoxybacillus* and *Geobacillus*. We isolated 20 thermophilic species from fouling samples of two dairy-production plants and screened for their ability to form biofilms. We identified three strains with relatively high biofilm-forming capacities and sequenced their genomes: *Geobacillus thermoglucosidans* TNO-09.020 (1), *Anoxybacillus flavithermus* TNO-09.006 (this work), and *Geobacillus stearothermophilus* TNO-09.008 (unpublished data).

The isolated strain *A. flavithermus* TNO-09.006 showed significant planktonic growth and biofilm-forming capacity at high temperatures (43 to 62°C; optimum at 57°C) under lab conditions (data not shown). This finding allows studies on the development of biofilm formation with the purpose of preventing the contamination of spores in end products.

The present view on genomic diversity, metabolic diversity, and adaptation of *A. flavithermus* to a dairy environment is limited, as only the genome of the strain *A. flavithermus* WK1 has been reported to date. This strain has been isolated from a wastewater drain at the Wairakei geothermal power station in New Zealand (2).

Genome sequencing was performed by GATC Biotech (Konstanz, Germany) using 454 Life Sciences GS20 pyrosequencing (1 M titanium; 454-Life Sciences, Roche). This sequence produced 181,707 reads totaling 72 Mb (average read length, 396 bp). In addition, Illumina mate pair sequencing was conducted (5,819,367 paired reads of 50 bp per read, 582 Mb). Assembly into contigs was performed using a whole-genome shotgun (WGS) assembler and SSPACE (3), which is a stand-alone program for scaffolding preassembled contigs using paired-end read data. A pseudoassembly was created by mapping the scaffolds to the most-similar complete genome sequence, that of *A. flavithermus* WK1 (GenBank accession no. CP000922.1) (2). A total of 82 gaps were filled using the consensus reads from PacBio *RS* sequencing (GATC Biotech) (5,402 reads; 7,857,232 bp; average read length, 1,456 bp). In short, the reads were mapped against the pseudoassembly using BLAST (PMID2231712) and Muscle (15034147), followed by manual inspection.

Automatic open reading frame (ORF) calling and annotation were performed using Integrated Services for Genomic Analysis (ISGA). Manual curation of ORFs was conducted using Artemis (4) and Artemis Comparison Tool (ACT) (5) by comparison with the reference *A. flavithermus* WK1 genome. Improved manual annotation was performed using Pfam (6), InterProScan (7), and NCBI BLAST. The genome of *A. flavithermus* TNO-09.006 consists of a single chromosome (2.65 Mb; 42% G+C content) in 1 scaffold containing 68 contigs; no plasmids were found. The chromosome contains approximately 2,761 protein-encoding genes, 53 tRNA-encoding genes, and 7 rRNA-encoding operons (usually at contig boundaries). As the genome of *A. flavithermus* TNO-09.006 is our second reported genome of a thermophilic isolate from a dairy-processing environment, a more detailed analysis of this genome and a comparative analysis with other thermophilic isolates will provide further insight into the specific properties related to the adaptation of these strains to the dairy-processing environment.

### Nucleotide sequence accession number. 

The complete genome of *A. flavithermus* TNO-09.006 has been deposited in GenBank under the accession no. AMCM00000000.
